# Apart From Rhoptries, Identification of *Toxoplasma gondii's O*-GlcNAcylated Proteins Reinforces the Universality of the *O*-GlcNAcome

**DOI:** 10.3389/fendo.2018.00450

**Published:** 2018-08-20

**Authors:** Moyira Osny Aquino-Gil, Mattis Kupferschmid, Hosam Shams-Eldin, Jörg Schmidt, Nao Yamakawa, Marlène Mortuaire, Frédéric Krzewinski, Stéphan Hardivillé, Edgar Zenteno, Christian Rolando, Fabrice Bray, Eduardo Pérez Campos, Jean-François Dubremetz, Yobana Perez-Cervera, Ralph T. Schwarz, Tony Lefebvre

**Affiliations:** ^1^Univ. Lille, CNRS, UMR 8576, UGSF, Unité de Glycobiologie Structurale et Fonctionnelle, Lille, France; ^2^Instituto Tecnológico de Oaxaca, Tecnológico Nacional de México, Oaxaca, Mexico; ^3^Centro de Investigación Facultad de Medicina UNAM-UABJO, Facultad de Odontología, Universidad Autónoma Benito Juárez de Oaxaca, Oaxaca, Mexico; ^4^Laboratory of Parasitology, Institute for Virology, Philipps-University, Marburg, Germany; ^5^Facultad de Medicina de la Universidad Nacional Autónoma de México, Mexico City, Mexico; ^6^CNRS, MSAP USR 3290, FR 3688 FRABIO, FR 2638 Institut Eugène-Michel Chevreul, Université de Lille, Lille, France; ^7^Unité Mixte de Recherche 5235, Dynamique des Interactions Membranaires Normales et Pathologiques, Université Montpellier, Montpellier, France

**Keywords:** *T. gondii*, *O*-GlcNAcome, *O*-GlcNAcylation, proteomics, toxoplasmosis, rhoptries

## Abstract

*O*-linked β-N-acetylglucosaminylation or *O*-GlcNAcylation is a widespread post-translational modification that belongs to the large and heterogeneous group of glycosylations. The functions managed by *O*-GlcNAcylation are diverse and include regulation of transcription, replication, protein's fate, trafficking, and signaling. More and more evidences tend to show that deregulations in the homeostasis of *O*-GlcNAcylation are involved in the etiology of metabolic diseases, cancers and neuropathologies. *O*-GlcNAc transferase or OGT is the enzyme that transfers the N-acetylglucosamine residue onto target proteins confined within the cytosolic and nuclear compartments. A form of OGT was predicted for *Toxoplasma* and recently we were the first to show evidence of *O*-GlcNAcylation in the apicomplexans *Toxoplasma gondii* and *Plasmodium falciparum*. Numerous studies have explored the *O*-GlcNAcome in a wide variety of biological models but very few focus on protists. In the present work, we used enrichment on sWGA-beads and immunopurification to identify putative *O*-GlcNAcylated proteins in *Toxoplasma gondii*. Many of the proteins found to be *O*-GlcNAcylated were originally described in higher eukaryotes and participate in cell shape organization, response to stress, protein synthesis and metabolism. In a more original way, our proteomic analyses, confirmed by sWGA-enrichment and click-chemistry, revealed that rhoptries, proteins necessary for invasion, are glycosylated. Together, these data show that regardless of proteins strictly specific to organisms, *O*-GlcNAcylated proteins are rather similar among living beings.

## Introduction

Most of the proteins are subjected to covalent chemical modifications that occur co- or post-translationally. Post-translational modifications (PTMs) are very diverse and consist in the addition—usually enzymatically—of simple or complex (e.g., peptides, proteins, glycosylphosphatidylinositol) groups, or in the proteolytic cleavage to enlarge the complexity of the proteome (1). To date, many hundreds of PTMs have been described among which are found a wide variety of glycosylations including the *O*-linked β-N-acetylglucosaminylation (*O*-GlcNAcylation). *O*-GlcNAcylation is a modification of cytosolic, nuclear and mitochondrial proteins by a single residue of N-acetylglucosamine transferred from UDP-GlcNAc which is provided by the hexosamine biosynthetic pathway (HBP). *O*-GlcNAcylation cycles on and off targeted proteins, and the addition and hydrolysis of the GlcNAc moiety is respectively controlled by OGT (*O*-GlcNAc transferase) and OGA (*O*-GlcNAcase) (2, 3). The functions managed by *O*-GlcNAcylation abound as testified by the non-exhaustive list of processes in which the PTM is involved: cell signaling, adaptation to stress, epigenetics, gene expression, protein synthesis and degradation, glycolysis, glucose sensing, enzymatic activity, circadian clock, immune response [for recent reviews see (3–6)]. Regulation of protein-to-protein interactions by *O*-GlcNAcylation (1, 7) directly or in close cooperation with other PTMs (1) seems to be at the nexus of such diversity of functions. Surprisingly, it was found that beyond protein *O*-GlcNAcylation, OGT also proteolytically processes the cell cycle regulator HCF-1 (Host cell factor-1) into a mature form through a glutamate glycosylation dependent manner (8, 9). Deregulations in *O*-GlcNAcylation cycling were reported in different human pathologies: cancers, type-2 diabetes, cardiovascular and neuronal disorders (10).

While extensively studied in cultured cell lines and animal models, few investigations have been reported regarding *O*-GlcNAcylation in intracellular parasites. In the early nineties, Dieckman-Schuppert and collaborators documented, for the very first time, *O*-GlcNAcylation in apicomplexans (11). Analyses of erythrocytes infected with *Plasmodium falciparum* indicated that parasite's *O*-glycans exhibiting terminal GlcNAc were *O*-GlcNAc. Ten years were necessary to identify MSP1 (Merozoite Surface Protein-1) expressed and GPI-anchored at the cell surface of *P. falciparum* to bear *O*-GlcNAc moieties (12). Nevertheless, due to its extracellular localization, MSP1 *O*-glycosylation is likely related to the extracellular glycosylation and not to the nucleocytoplasmic *O*-GlcNAcylation. But, in the late 2000's, OGTs expressed by *Cryptosporidium parvum*, that is phylogenetically close to *Plasmodium* and *Toxoplasma gondii*, and by the minimalist protist *Giardia lamblia* were characterized (13). Later, our team revealed that *T. gondii* unambiguously expresses the nuclear and cytoplasmic modification and strongly suggested its occurrence in *P. falciparum* (14). Very recently, we identified 13 *O*-GlcNAcylated proteins expressed in *P. falciparum* among which Hsp70, alpha-tubulin, actin and glycolytic enzymes (15). In the present report, we used similar approaches and revealed part of *Toxoplasma gondii*'s *O*-GlcNAcome which, except rhoptry proteins, is very close to those found not only in *Plasmodium falciparum* but also in higher eukaryotes. Together, these findings tend to demonstrate that a large part of the *O*-GlcNAcome is universal, that is conserved through species.

## Materials and methods

### Culture and preparation of the parasites

*T. gondii* grown in African green monkey kidney cells (Vero cells, ATCC CCL-81) or in human foreskin fibroblast (HFF, ATCC SCRC-1041), were cultured in DMEM (Dulbecco's Modified Eagle Medium, Gibco BRL), and supplemented with 10% (v/v) fetal calf serum (FCS, Gibco), 2 mM glutamine, 100 units/mL penicillin, and 0.1 mg/mL streptomycin. Parasites (5 × 10^7^) were added to confluent monolayer of cells (175 cm^2^), harvested after being cultivated for 72 h, and liberated from their host cells using a Mixer Mill homogenizer (Retsch). The suspension was run through a 20 mL glass-wool column to remove cellular debris. The purity of the tachyzoite suspension was monitored microscopically. Cell lines and parasites were routinely tested for *Mycoplasma* contamination. To control the efficiency of parasite purification, *T. gondii* cells liberated from their host cells were mixed with homogenized Vero cells, and the mixture was purified using glass-wool columns as described previously (16).

### Protein extraction

The parasites were lysed on ice in the following buffer: 10 mM Tris/HCl, 150 mM NaCl, 1 mM EDTA, 1% (v/v) Triton X-100, 0.5% (w/v) sodium desoxycholate, 0.1% (w/v) SDS, pH 7,4. After vigorous mixing, the lysate was centrifuged at 20,000 g for 10 min at 4°C. The pellet was discarded and the supernatant saved for further analyses. For sWGA (succinylated-Wheat Germ Agglutinin)-beads enrichment, parasites were lysed in homogenization buffer containing 10 mM Tris/HCl, 1 mM EDTA, 1 mM EGTA, 0.5% (v/v) Triton X-100, pH 7.5. A cocktail of proteases inhibitors was added freshly to buffers before use.

### SDS-PAGE and western blot

Proteins were resolved on 8 or 10% SDS-PAGE and either silver stained or electroblotted onto nitrocellulose membrane. Equal loading and transfer efficiency were checked using Ponceau red staining. Membranes were saturated in 5% (w/v) non-fatty milk in Tris buffered Saline (TBS)-Tween (15 mM Tris, 140 mM NaCl, 0.05% (v/v) Tween) for 1 h or in 5% (w/v) bovine serum albumin (BSA) in TBS-Tween overnight. The primary antibodies anti-*O*-GlcNAc, anti-tubulin and anti-rhoptries were incubated overnight at 4°C. Then nitrocellulose membranes were washed three times for 10 min each in TBS-Tween and incubated with horseradish peroxidase-labeled secondary antibodies for 1 h or with avidin-HRP for 45 min. HRP-rPVL was used as previously described (17). Finally, three washes of 10 min each were performed with TBS-Tween and detection was carried out with enhanced chemiluminescence (GE Healthcare).

### Succinylated-WGA protein enrichment

Prior to sWGA-beads enrichment, 500 μg of proteins were incubated with sWGA beads overnight in a buffer containing 10 mM Tris/HCl, 100 mM NaCl, 0.4% (w/v) sodium deoxycholate, 0.3% (w/v) SDS and 0.2% (w/v) Nonidet P-40. Beads were washed four times using the same buffer without and then with free GlcNAc. After boiling in Laemmli buffer, proteins were resolved by SDS-PAGE.

### Labelling of *O*-GlcNAcylated proteins by click-chemistry

Labelling of *O*-GlcNAc-bearing proteins by GalNAz and biotin alkyne was done using the Click-it *O*-GlcNAc enzymatic labeling system and the Click-it Glycoprotein detection kit (Biotin alkyne) according to the manufacturer's instructions (Fischer Scientific) (18). Bovine α-crystallin was used as a positive control. After labeling, proteins were precipitated using the methanol/chloroform protocol and resuspended in 50 μL of Tris/HCl pH 8.0 containing 0.1% (w/v) SDS. 700 μL of enrichment buffer (1% (v/v) Triton X-100 and 0.1% (w/v) SDS in PBS) was added to the sample before incubating with 50 μL of avidin-coupled beads (1 h, 4°C). Avidin-bound proteins were collected, washed three times with enrichment buffer, resuspended in Laemmli buffer and boiled. Controls of labeling and enrichment followed the same procedure except that the chemical labeling with UDP-GalNAz was omitted.

### Mass spectrometry

Proteins from *T. gondii* were enriched using either sWGA as specifed above or using the anti-*O*-GlcNAc antibody RL2 as described in Dehennaut et al. (18). Bound-proteins were resolved by SDS-PAGE and silver stained or Coomassie blue stained. Specific bands were excised from the gels. The pieces of gel were dehydrated using a 50:50 dilution of 50 mM Ammonium Bicarbonate (Bic) (HPLC Grade, Prolabo) and Acetonitrile (ACN) (Sigma A) followed by 100% ACN. They were then reduced in 20 mM DTT (Sigma) in 50 mM Bic and alkylated in 100 mM iodoacetamide (Bio-Rad) in 50 mM Bic. After washes with ACN/Bic, the bands were digested with 100–200 ng Trypsin Gold (Promega) in 25 mM Bic. Extraction was done using 45% (v/v) ACN and 10% (v/v) formic acid (Sigma). Extracted peptides were purified using C_18_-Zip-Tip cones using 0.1% (v/v) Formic Acid for washes and 50:50 ACN/0.1% (v/v) Formic Acid for elution. The nano-LC MS/MS analysis was performed on a HPLC system with two LC-20AD nano-flow LC pumps, an SIL-20 AC auto-sampler and an LC-20AB micro-flow LC pump (Shimadzu, Kyoto, Japan) connected to an ion-trap mass spectrometer (amaZon ETD, Bruker Daltonics, Bremen, Germany) equipped with a Captive Spray ion source. Hystar (Version 3.2, Bruker Daltonics, Bremen, Germany) was used to couple and control Shimadzu CBM-20A module (Shimadzu, Kyoto, Japan) for MS acquisition for all experiments.

Trapping and desalting of the peptides was carried out on nano trapping column (Zorbax 300SB-C18, 5 μm, 0.3 × 5 mm, Agilent) using 0.05% (v/v) trifluoroacetic acid solution for 10 min at a flow rate of 10 μL/min. After back-flushing from the trapping column, peptides were separated on a reversed-phase column, ACQUITY UPLC® M-Class Peptide BEH C18 Column (1.7 μm, 130 Å, 100 × 0.75 mm i.d., Waters) using an acetonitrile/0.1% (v/v) formic acid gradient from 15 to 50% (v/v) acetonitrile within 30 min. Fine tuning was achieved using the smart parameter setting (SPS) option for m/z 800, compound stability and trap drive level were set at 100%. Optimization of the Captive Spray source resulted in dry gas temperature, 150°C, dry gas, 3.0 L/min, capillary voltage, −1,200 V, end plate offset, 0 V. The MS1 and MS2 ion detection windows were set at m/z 100–1,700. The five highest no singly charged peaks in each MS1 spectrum were automatically fragmented through collision-induced dissociation (CID).

### Data processing

The LC-MS/MS data was analyzed using DataAnalysis 4.1 software (Bruker Daltonics) and the function Compound–sAutoMS(n) was used to generate 1,200 compound spectra. Generated data were exported as a mascot generic file. Protein identification through primary sequence database searching was performed using the MASCOT search algorithm (MASCOT free version; Matrix Science, London, UK). The following MASCOT settings were used: taxonomy: *Toxoplasma gondii*; database: SwissProt; enzyme: trypsin; fixed modifications: carbamidomethyl (C); variable modifications: oxidation (M, HW), deamidation (NQ), phosphorylation (ST), pyro-Glu (N-term E), and HexNAc (ST); maximum missed cleavages: 1; MS1 peptide tolerance: 0.6 Da; MS/MS tolerance: 0.6 Da.

### Immunofluorescence microscopy

Immuno-labeling of parasites was done in tube (solution) and then were observed on glass slides. Purified *T. gondii* were fixed in 3% (m/v) of paraformaldehyde in cold PBS for 15 min and subsequently washed with PBS. Unreacted aldehyde groups were quenched with a solution of 50 mM ammonium chloride for 10 min. After several washes with PBS, cells were permeabilized with 0.1% (v/v) Triton-X100 for 5 min. Nonspecific sites were blocked with goat serum. *T. gondii* were then incubated for 30 min with a dilution of 1:100 in a 10% (v/v) goat serum solution (in PBS) of anti-*O*-GlcNAc antibodies (RL2), complemented or not with free GlcNAc. After 3 washes with PBS, parasites were incubated with fluorescein isothiocyanate (FITC) antibodies (dilution 1:50). *T. gondii* were visualized using an Axioplan 2 imaging microscope (Zeiss, Jena, Germany) and an Axio Cam HRc camera (AxioVision; Zeiss).

## Results

### Visualization of the *Toxoplasma gondii O*-GlcNAcome

**Figure 1:**
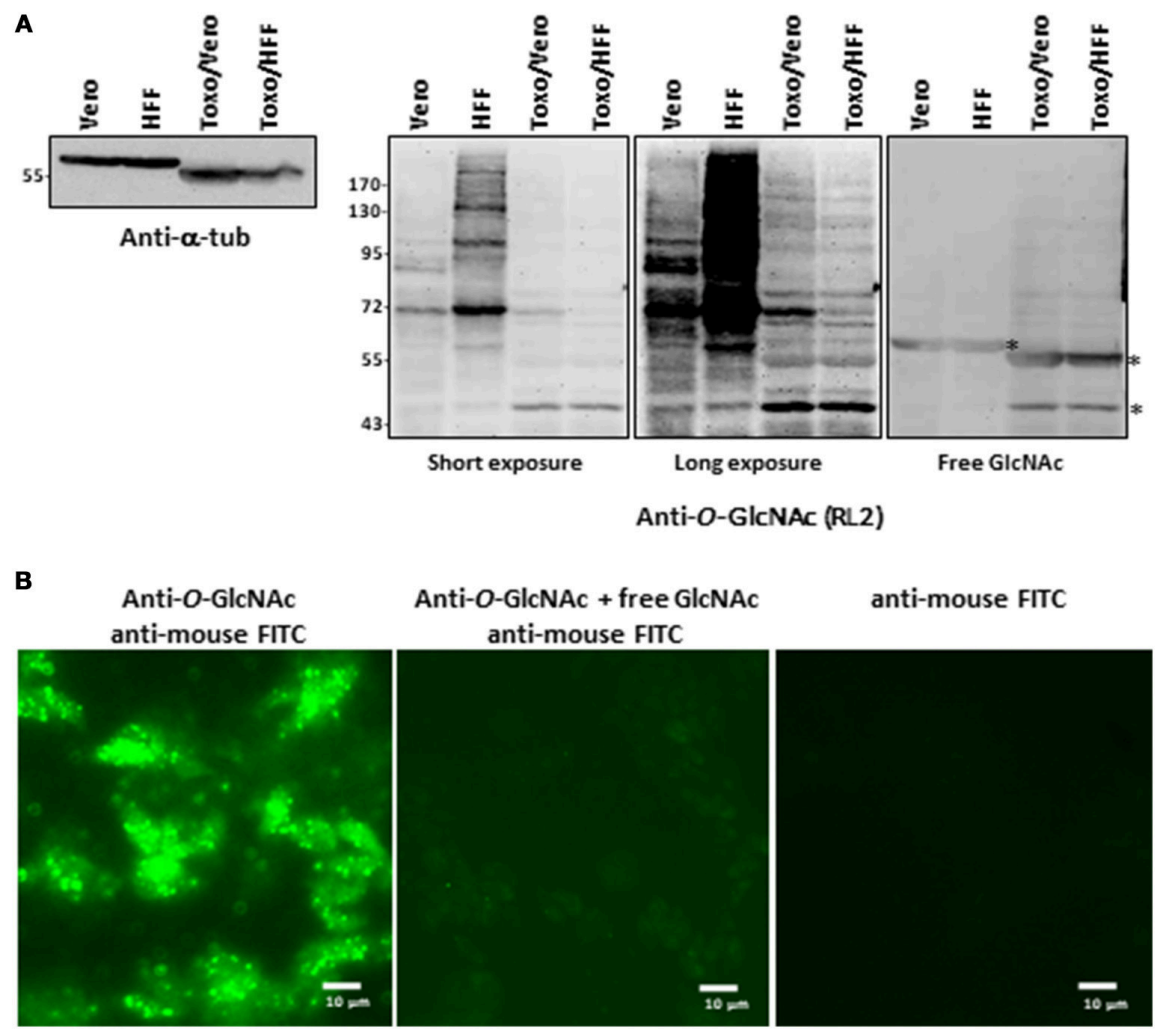
**(A)** Parasites were produced either in Vero cells or HFF. After homogenization, proteins were resolved by SDS-PAGE and electroblotted onto nitrocellulose and probed with anti-alpha-tubulin antibody or with RL2 anti-*O*-GlcNAc antibody alone or in conjunction with free GlcNAc to analyse their purity and their *O*-GlcNAcylation content respectively. **(B)** Parasites were stained with anti-*O*-GlcNAc antibody as primary antibody and FITC-labeled anti-mouse as secondary antibody and imaged by fluorescent microscopy. Controls were done by co-incubation of anti-*O*-GlcNAc antibody with free GlcNAc and by staining with secondary antibodies alone.*, unspecific bands. HFF, human foreskin fibroblast; T.g., *Toxoplasma gondii*; FITC, Fluorescein isothiocyanate.

*T. gondii* used in this study were prepared by infecting either Vero cells or HFF (human foreskin fibroblasts). The absence of contamination of parasite preparations by host cells was unequivocally attested by microscopy examination and anti-α-tubulin staining (Figure 1A). α-tubulin is expressed in Vero cells, HFF and *T. gondii* but the difference of molecular weight observed between the two cell types and the parasite asserts that there was no contamination during *T. gondii* purification. Host cells and *T. gondii* proteins were also Coomassie brilliant blue stained and the different protein profiles compared (data not shown).

Distinct approaches based on antibodies, click-chemistry and lectin-beads enrichment were used to visualize the occurrence of *O*-GlcNAcylation in *T. gondii*. First, protein extracts prepared from parasites grown either in Vero or in HFF cells and resolved by SDS-PAGE and electrotransferred onto nitrocellulose membranes were stained with the anti-*O*-GlcNAc antibody RL2—originally raised against nuclear pore proteins (19) (Figure 1A). We found that the parasites display distinct *O*-GlcNAcylated proteins profiles, which differ from those of Vero or HFF cells indicating further absence of any cross-contamination by host cells. Incubation of the anti-*O*-GlcNAc antibody with 0.5 M of free N-acetylglucosamine abolished most of the signal, asserting the specificity of the reaction. *O*-GlcNAcylation was also detected by immunofluorescent microscopy by incubating the purified parasites with the RL2 antibodies alone or in conjunction with free GlcNAc, or with the secondary antibodies alone (Figure 1B).

**Figure 2:**
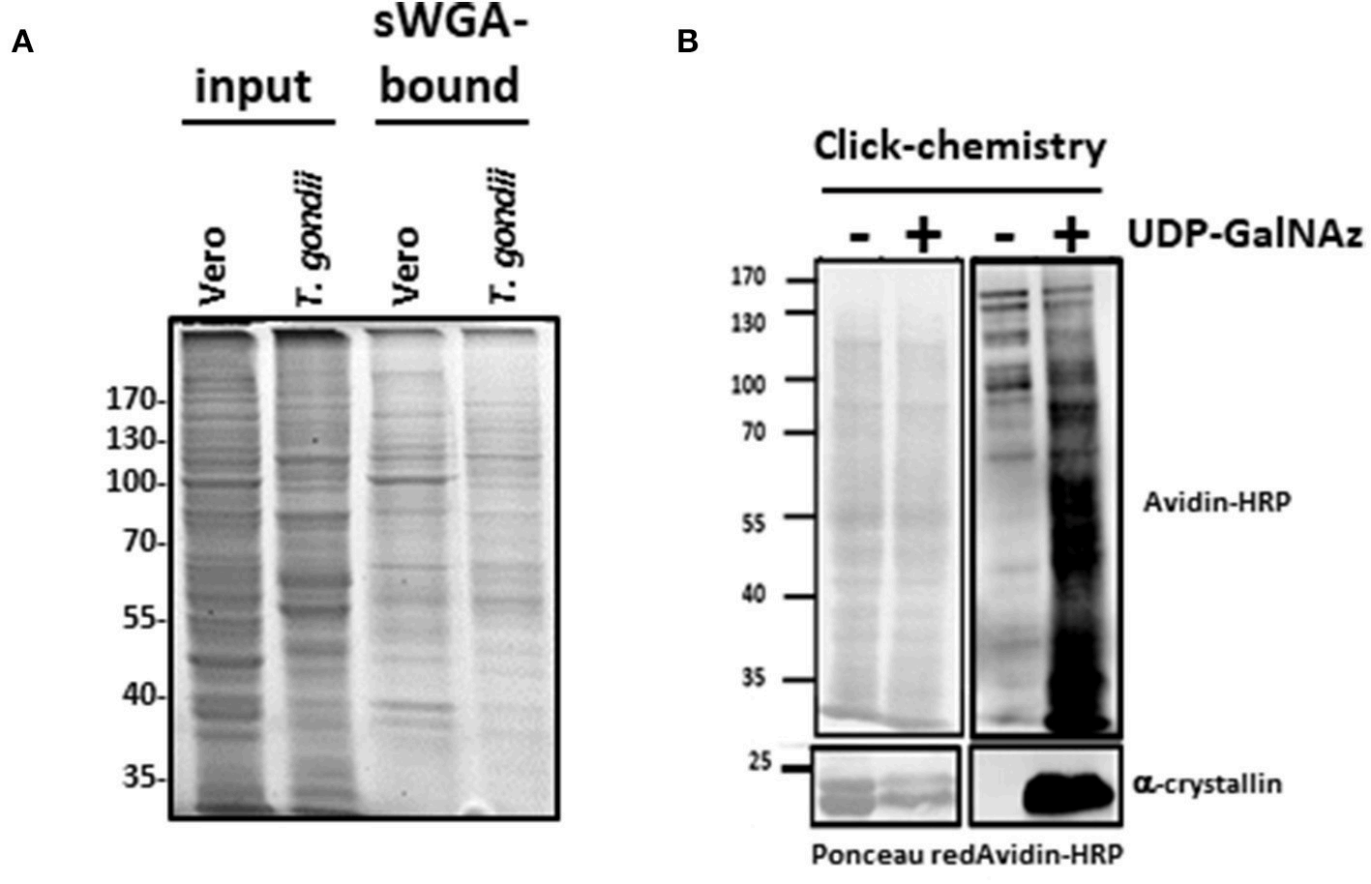
**(A)** *O*-GlcNAc-bearing proteins from Vero cells (host cells) and *T. gondii* were enriched by sWGA-beads. Gels were brilliant blue stained. **(B)** *T. gondii* proteins were analyzed after labeling of the *O*-GlcNAc-bearing proteins by GalNAz and biotin alkyne using the Click-it™ *O*-GlcNAc enzymatic labeling system and the Click-it™ Glycoprotein detection kit as described in the Material and methods section. After labeling, *T. gondii* samples were separated by SDS-PAGE and Western blot was performed using HRP-labeled avidin to assess presence of the labeled *O*-GlcNAc-proteins. Equal loading was controlled by staining proteins with Ponceau red. Alpha-crystallin was used to control labeling efficiency.

Parasite protein extracts were enriched on sWGA-coupled beads (Figure 2A) or processed through a Click-chemistry procedure (Figure 2B). sWGA is the plant lectin WGA chemically modified with a succinyl group to prevent the lectin to bind to sialic acids. Therefore, when compared to unmodified WGA, sWGA interacts only with GlcNAc residues (20). sWGA was immobilized on beads and incubated with *T. gondii* extracts. After intensive washes (not shown), bound fractions were eluted, resolved by SDS-PAGE and brilliant blue stained as described in Kupferschmid et al. (15). Vero cells were processed according to the same procedure and compared to parasites: distinct profiles of sWGA-bound proteins can be observed (Figure 2A). Regarding the Click-chemistry approach, we used the Click-iT TM system to biotin-label *O*-GlcNAcylated proteins. Clicked proteins were detected with avidin-labeled HRP (Figure 2B). This technique is highly sensitive as attested by the strong signal observed after staining the clicked-proteins with peroxidase-labeled avidin. A negative control, in which UDP-GalNAz has been omitted, was used to differentiate endogenous biotinylation from biotin-labeling. Nevertheless, GalNAz can be grafted to any terminal GlcNAc, therefore this approach alone is not sufficient to discriminate strictly *O*-GlcNAcylation. It is why it was necessary to combine distinct tools (anti-*O*-GlcNAc antibody, sWGA and click-chemistry) to confirm that *T. gondii*'s proteome is largely *O*-GlcNAcylated, and thus, like many other species, possesses its own *O*-GlcNAcome (Figures 1, 2) in accordance with previous reports (14, 21) RL2.

### Exploration of *T. gondii*'s *O*-GlcNAcome by two independent approaches

To increase the chance to identify *O*-GlcNAcylated proteins expressed in *T. gondii*, we conducted experiments through two distinct approaches. First, the anti-*O*-GlcNAc antibody RL2 was used to enrich *O*-GlcNAcylated proteins, control experiment was done with non-immune immunoglobulin. The chemically modified plant lectin sWGA was used as a second approach. Bands of the proteins enriched using either the anti-*O*-GlcNAc antibody or sWGA were excised from the gels. Proteins were digested with trypsin and analyzed by tandem mass spectrometry as detailed in the materials and methods section. The protein identification was performed using the Mascot search engine against the SwissProt database restricted to *T. gondii* taxonomy. This allowed the identification of numerous proteins (Supplementary Data Table 1): 65.5% using sWGA and 34.5% using the anti-*O*-GlcNAc antibody, fifty proteins being identified by both approaches. Identified proteins can be classified according to their functions and take part in ribosome biogenesis, RNA binding and processing, intracellular transport, signal transduction, lipids metabolism, glycolysis, tricarboxylic acids cycle, organization of cytoskeleton, as well as many other cellular functions.

**Figure 3:**
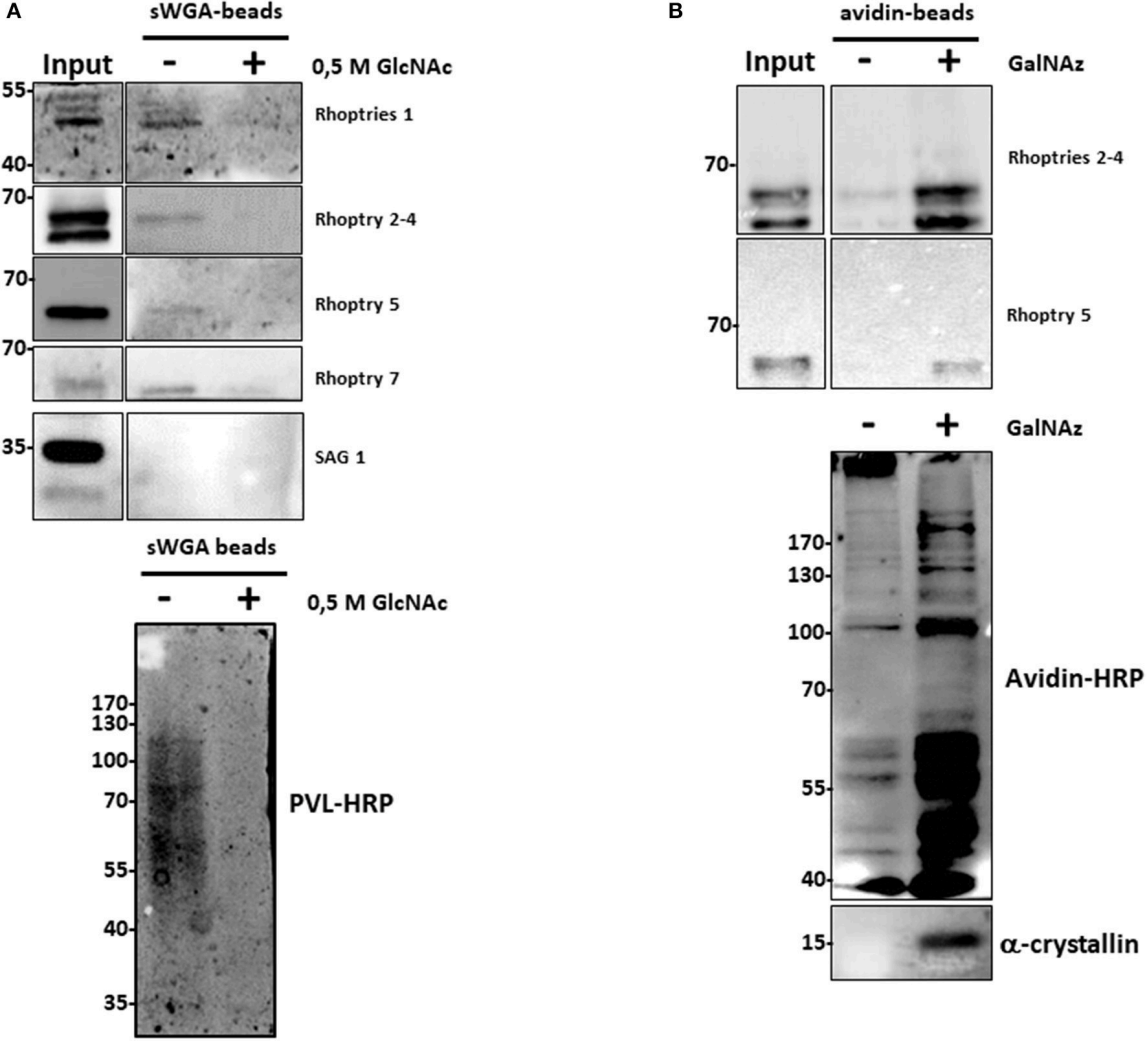
**(A)** After enrichment on sWGA-beads as described in the materials and methods section, bound proteins were submitted to SDS-PAGE and Western blot. Membranes were probed with different anti-rhoptries antibodies, anti-SAG 1 antibody or with the lectin PVL-HRP. Specificity of PVL-HRP was confirmed by incubation in presence of free GlcNAc. **(B)** *T. gondii* proteins were enzymatically labeled with GalNAz and then chemically with avidin. Proteins were enriched by avidin-beads and samples were resolved by SDS-PAGE and western blotted. Membranes were probed with indicated anti-rhoptries antibodies. Control was performed by staining clicked-proteins with avidin-HRP. Alpha-crystallin was used as a control of labeling.

Surprisingly, several proteins involved in the infection process of *T. gondii*, called rhoptries, were identified by both approaches (sWGA and anti-*O*-GlcNAc antibodies). To confirm that rhoptries are indeed *O*-GlcNAcylated proteins, we tested antibodies specifically directed against this group of proteins (Figure 3). Lysates from *T. gondii* were either enriched using sWGA-beads or processed through the click-chemistry approach followed by enrichment on avidin-beads, resolved by SDS-PAGE and analyzed by Western blot. Rhoptries 1, 2, 5, and 7, and rhoptries 2, 4, and 5 were recovered by sWGA (Figure 3A) or click-chemistry (Figure 3B) enrichment respectively. SAG 1 was used as a negative control since it is known that its glycans exhibit terminal mannose (22). These two methods associated to our proteomic analyses revealed the *O*-GlcNAcylation of rhoptries.

## Discussion

Since its discovery in the eighties (23, 24), *O*-GlcNAcylation has not ceased to arouse the interest of the scientific community. First due to its ubiquitous feature since it is widespread in a large panel of living beings, and secondly because it affects countless proteins, confined within the cytosolic, nuclear and mitochondrial compartments, involved in the regulation of many fundamental biological processes (3, 25). In a recent review, we came back on the different living organisms expressing their own OGT or hijacking the *O*-GlcNAcylation machinery of host cells (2), and stated that OGT emerged as a master regulator of cell homeostasis.

During the last years, *O*-GlcNAcomes of diverse biological models (cell lines, tissues, whole organisms) were explored (1, 15, 18, 25–29). However, very few studies have described *T. gondii*'s glycome and virtually none have investigated its *O*-GlcNAcome. *T. gondii* is an obligate intracellular parasite identified in 1908 in the North African rodent gundi (*Ctenodactylus gundi*) by Nicolle and Manceaux (30). Like other apicomplexans (*Plasmodium* and *Theileria*), *T. gondii* is responsible for debilitating diseases in humans and warm-blooded animals. The worldwide zoonosis toxoplasmosis is one of these major infectious diseases. In France, its seroprevalence in pregnant women is near 54% (31) and it is estimated that a third of human population is infected worldwide. In the majority of the cases, the infection has no serious consequences for immunocompetent patients; nevertheless, a reactivation of the latent parasite in immune compromised individuals may cause encephalitis. It is also noteworthy that cerebral toxoplasmosis is a major cause of morbidity and mortality among AIDS patients. Therefore, fighting the infection by *T. gondii* remains a major challenge to public health, and anything that can contribute to increase the current knowledge on the biology of the parasite can be considered as breakthroughs and opportunities to open novel therapeutic solutions.

**Figure 4:**
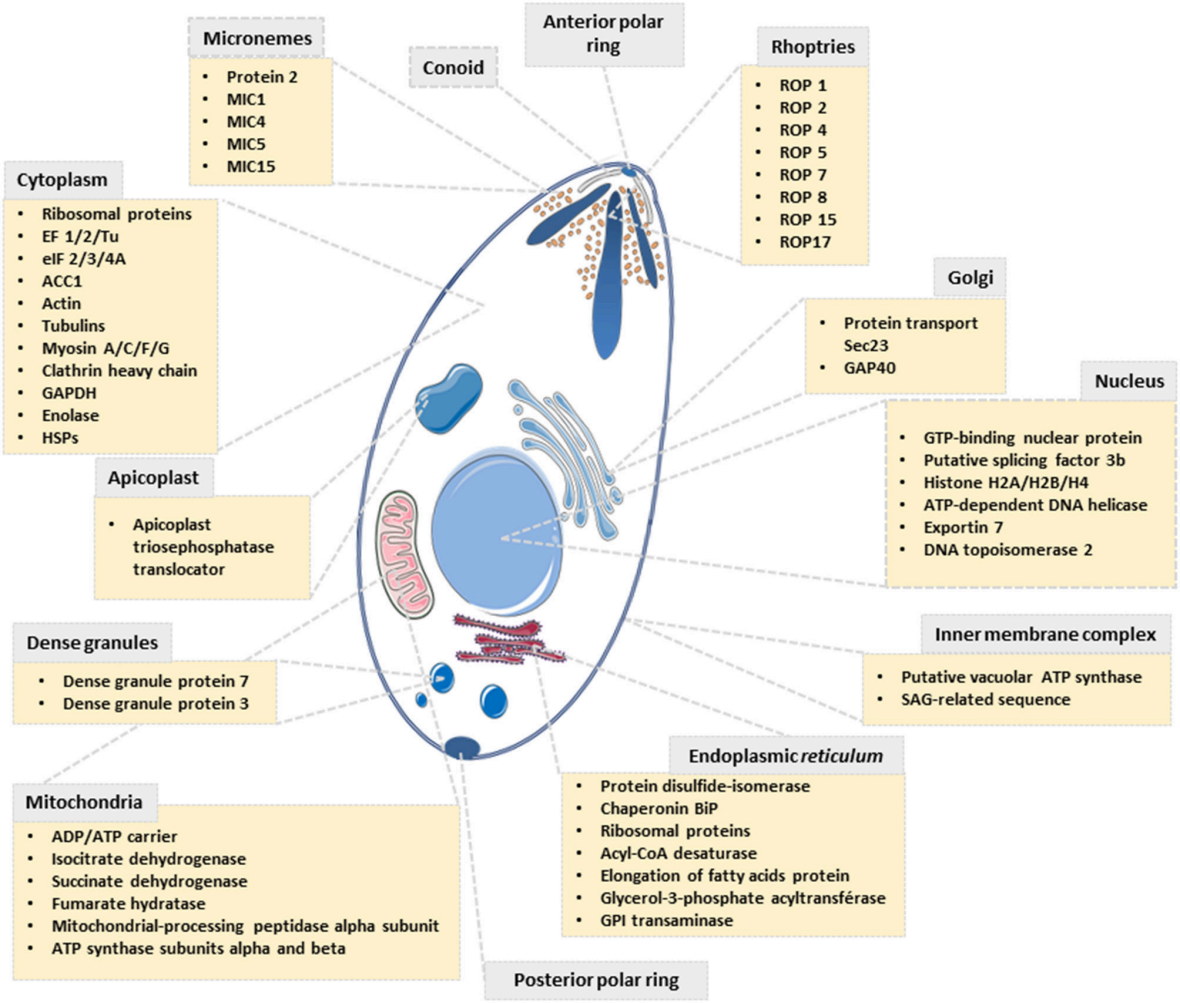
Scheme depicting the subcellular localization of proteins identified to be *O*-GlcNAcylated in *T. gondii*.

**Figure 5:**
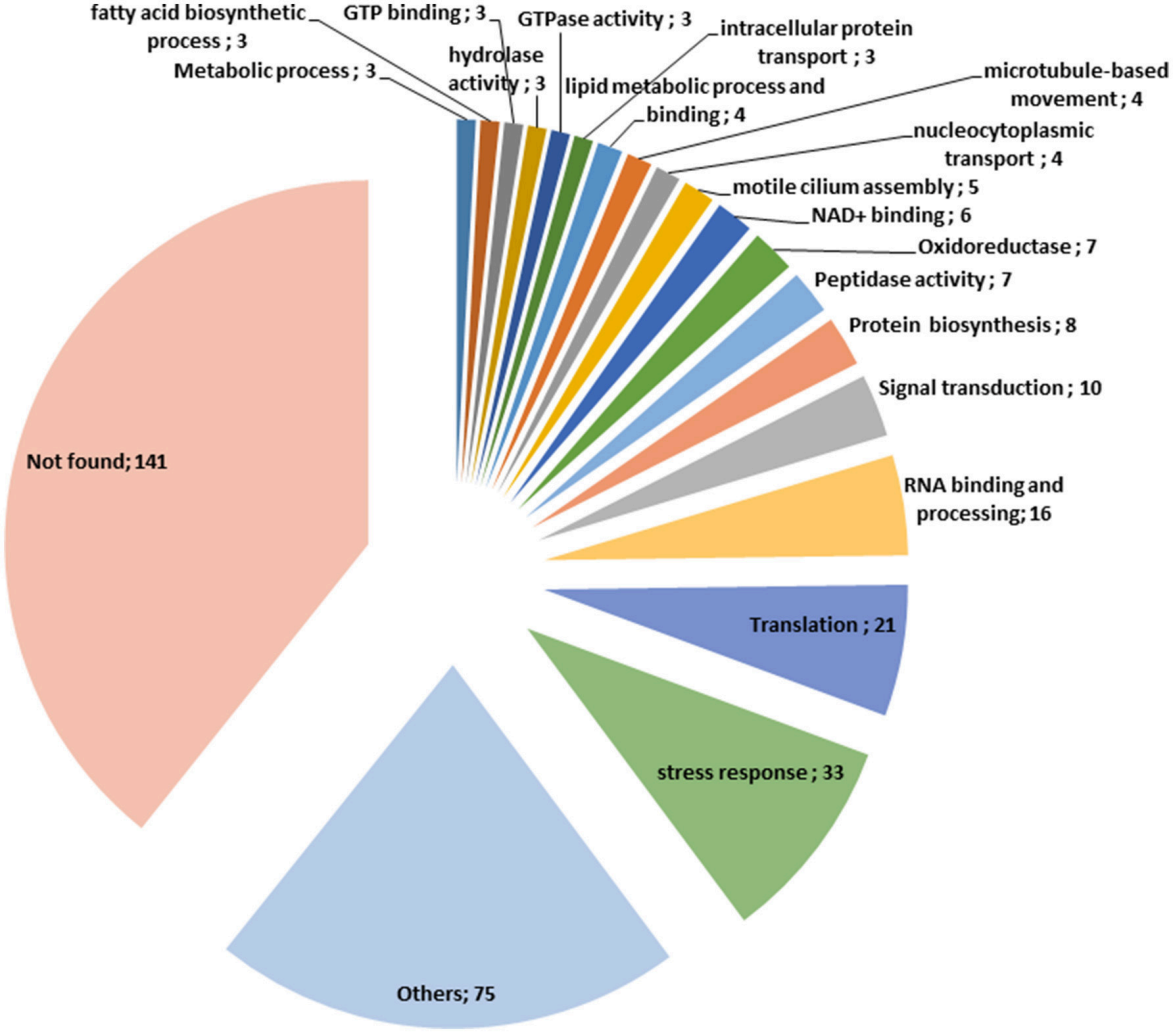
Classification of the *O*-GlcNAcylated proteins found in this study according to their function.

**Figure 6:**
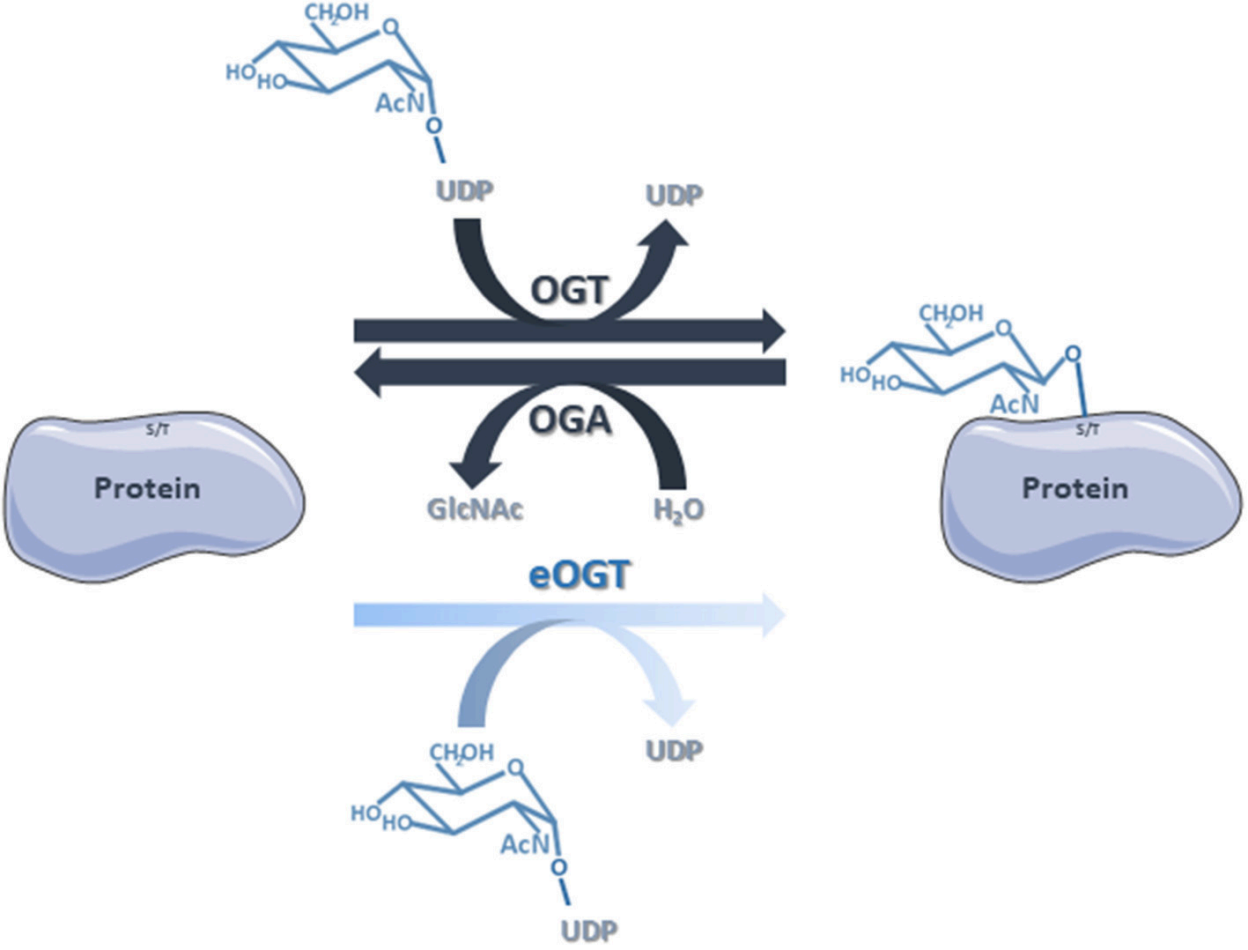
The classical *O*-GlcNAcylation cycling is managed by OGT that transfers the GlcNAc group from the donor UDP-GlcNAc to the target proteins, and OGA that removes the residue by hydrolysis. Nevertheless, we do not rule out that some of the proteins identified in this study are substrates of eOGT. eOGT is located in the endoplasmic *reticulum* where it glycosylates secreted and membrane proteins, and is suggested to modify proteins confined within intracellular compartments. In contrast to *O*-GlcNAcylation driven by OGT/OGA, it is unlikely that eOGT-catalyzed *O*-GlcNAcylation is versatile.

**Figure 7:**
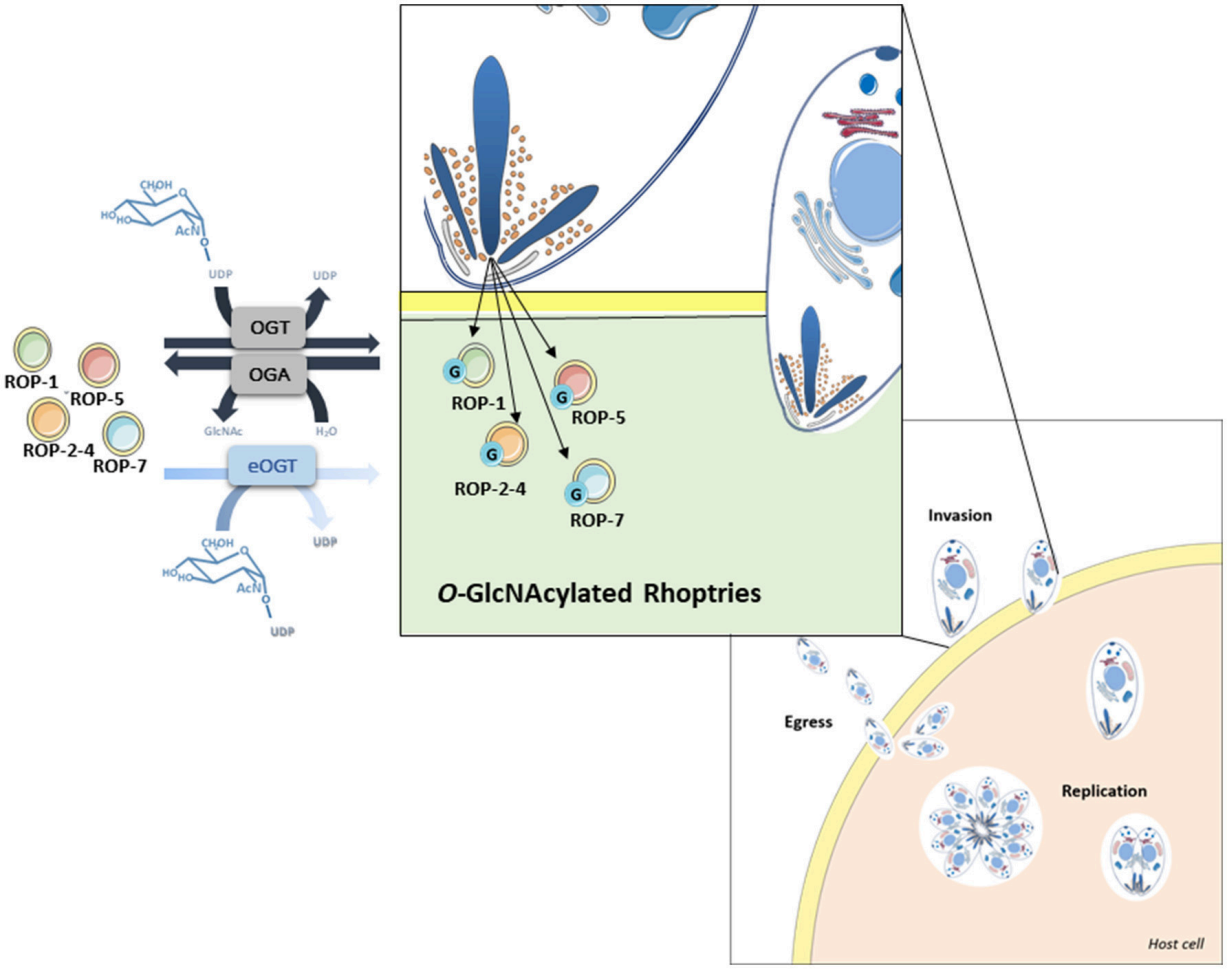
*O*-GlcNAcylated rhoptries in *Toxoplasma gondii* and their putative involvement in infection.

Our proteomic analyses revealed the *O*-GlcNAcylation of many proteins involved in a large panel of biological functions (heat shock proteins, glycolytic enzymes, ribosomal proteins, actors of protein synthesis and lipid metabolism) (Figures 4, 5). Most of the *O*-GlcNAcylated proteins identified in this study were previously reported to be *O*-GlcNAc proteins in other models reinforcing the ubiquitous role of *O*-GlcNAcomes found in diverse species. More originally, we highlighted proteins involved in the infection process: rhoptries, microneme proteins and dense granules, all part of secretory organelles and characteristic of the motility of apicomplexans (Figure 4). The two former are part of a specific apical complex that also includes a conoid that consists of tubulin fibers (32). Microneme proteins are synthesized by micronemes, specialized organelles involved in secretion, which support several key cellular processes such as invasion, motility and migration through host cells (33). Microneme proteins are first released during infection followed, thereafter, by a second wave of secretion that involves rhoptry proteins. Rhoptry proteins are trafficked from the endoplasmic *reticulum* to the Golgi apparatus, and then are addressed to secretory organelles. Therefore, it cannot be rule out that *O*-GlcNAcylation of rhoptries is performed by EOGT–for EGF (Epidermal Growth Factor) domain-specific OGT (34)–that controls a second form of *O*-GlcNAcylation affecting proteins of the secretory pathway (extracellular matrix proteins, extracellular region of transmembrane proteins) such as Notch receptors (Figure 6). A default in the activity of EOGT is responsible for the rare congenital disorder Adams-Oliver syndrome (35). Previously, two independent studies have reported glycosylation of rhoptries (21, 36) by conducting different strategies. Luo and collaborators used a combination of lectins (ConA, WGA and Jacalin) to identify 132 glycoproteins among which rhoptries and microneme proteins (36). Later, *T. gondii*'s glycoproteome has been investigated by the use of unnatural sugars through a bioorthogonal chemical reporter strategy (21). The function of the glycosylation of rhoptries (Figure 7) and micronemes in the infection remains unknown, but it should be of interest to determine whether interfering with their *O*-GlcNAcylation reduces motility and invasion of *T. gondii*.

*O*-GlcNAcylation levels correlate with nutrients status of the cell. UDP-GlcNAc, the substrate of OGT, is at the crossroad of many metabolic pathways (2). Diet may then affect the ability of *T. gondii* to infect host cells and further investigation on whether infectious process of the parasite depends on *O*-GlcNAcylation and/or nutrient is of great interest. It remains also to determine in a near future whether *O*-GlcNAcylation cycling regulates the fate of rhoptry proteins at the expression and degradation levels. Also, it is now well recognized that *O*-GlcNAcylation, like other PTMs, controls protein-protein interactions (1, 7) and the impact of *O*-GlcNAcylation dynamics on biogenesis of rhoptry organelle remains to be investigated.

This paper provides a large-scale analysis of putative *T. gondii*'s *O*-GlcNAcome. Understanding the role of the glycosylation of these proteins may help to elucidate mechanisms involved in invasion and cell life cycle (Figure 7), which would lead to improve drug therapy as well as to identify glycoproteins that may prove to be useful as therapeutic targets.

## Author contributions

MA-G, YP-C, RS, and TL developed the hypotheses. MA-G performed most of the biochemical experiments and interpreted the data. MK and MM performed experiments and gave important technical training to MA-G. SH gave important advices and help experimenting. HS-E and JS contributed to the culture of *T. gondii*. J-FD produced and provided antibodies against rhoptries. NY, FK, FB, and CR performed the mass spectroscopic analysis and provided the resulting data. TL, RS, EP, and YP-C initiated the project. MA-G, J-FD, YP-C, EZ, RS, and TL analyzed the results. TL drafted the manuscript. All authors contributed to refining the manuscript, read and approved the final version.

### Conflict of interest statement

The authors declare that the research was conducted in the absence of any commercial or financial relationships that could be construed as a potential conflict of interest.

## Supplementary material

Supplementary Data Table 1 List of the proteins identified in this study. Proteins identified by enrichment with sWGA are in green, and by enrichment with RL2 are in pink.
